# Supplementary material for: Extraction of driving behavior primitives considering driver expectation and vehicle dynamics
Source: PLoS One. 2025 Dec 31;20(12):e0340123. doi: 10.1371/journal.pone.0340123 (PMC12755792; doi:10.1371/journal.pone.0340123)
Supplement: Attachment — Submitted filename: renamed_d5373.docx [file pone.0340123.s004.docx]

**Dear reviewers and editors,**

We would like to express our sincere gratitude to you for your meticulous reading and valuable comments on our manuscript. Your feedback has been crucial for improving the quality of the manuscript and has provided new perspectives for our research. In accordance with your guidance, we have thoroughly revised the manuscript. We believe these revisions have significantly enhanced the quality of the manuscript and sincerely invite you to review our revised version. We have worked diligently to make this manuscript as strong as possible, but we recognize that there may always be room for improvement. We would be very grateful for any further comments or suggestions you might have.

Below is our point-by-point response to each of the reviewers’ comments.

# Reviewer #1

## Question 1

***Question:*** Although the abstract mentions improvements over the traditional LDA through the proposed VC-LDA approach, it lacks clarity on the evaluation metrics used and the nature of the dataset involved.

**Answer:** Thank you for your valuable feedback. We agree with the reviewer’s comment that the original abstract lacked clarity regarding the evaluation metrics and the nature of the dataset. To address this, we have thoroughly revised the abstract to incorporate these essential details, providing a more complete overview of our work. The revised abstract has been highlighted in the manuscript for your convenience.

***ABSTRACT:*** *Extracting meaningful driving behavior primitives is crucial for fine-grained behavior analysis. To develop primitives that more closely mirror the hierarchical nature of human driving, this paper introduces a novel framework and carried out on a 16 drivers’ dataset. The proposed method operates in two synergistic stages. First, for behavior segmentation, a multi-type feature space is constructed to capture both the objective vehicle motion states and the subjective driver expectations for vehicle performance. This space is then input into a Bayesian Model-based Agglomerative Sequence Segmentation (BMASS) model to achieve precise segmentation. Segment durations and the positioning of segmentation points serve as key metrics to assess the quality of behavior segmentation. Next, for primitive clustering, a Variable Coupling-based Latent Dirichlet Allocation (VC-LDA) method is proposed. The core innovation of VC-LDA lies in a features-coupling-aware discretization process. By considering the non-linear coupling and temporal asynchrony among features in the multi-type space, this process yields driving states with enhanced physical interpretability, providing a high-quality foundation for LDA clustering. Experimental results demonstrate that the VC-LDA model significantly outperforms the GMM-LDA, achieving a much lower perplexity and exhibiting higher intra-class compactness in topic-word distributions. This framework offers an automated and efficient pathway to understand and model driver behavioral patterns, providing valuable insights for the development of ADAS and AVs.*

## Question 2

***Question:*** While the research gap regarding driver expectation and vehicle dynamics is well identified, the study fails to properly address the underlying cognitive or theoretical models that justify these constructs.

**Answer:** Thank you for this insightful comment. You correctly point out that our initial manuscript lacked an explicit theoretical model to justify the link between driver expectations and vehicle dynamics. We acknowledge this as a significant oversight and have revised the paper to address it.

Our underlying hypothesis is that driving behavior can be understood through a hierarchical model. At the operational level, driving behavior are directly reflected in basic vehicle dynamics (e.g., velocity, acceleration, jerk), which are explicit observations of the vehicle motion state. However, at the tactical level, drivers make short-term decisions based on their expectations for multiple, often competing, performance objectives, such as safety, efficiency, economy, and comfort. It is this higher-level, expectation-driven decision-making that fundamentally shapes the observed vehicle dynamics at the operational level.

The “constructed features” in our study were designed to be quantitative proxies for these tactical-level expectations. By incorporating them alongside the basic features, we enable the BMASS algorithm to segment driving behavior not just on the basis of vehicle motion, but on the underlying driver intentions that govern that motion.

Following your recommendation, we have now formalized this cognitive hierarchy and added it to ***Section 3.1*** to provide a robust theoretical foundation for our multi-type feature space. For your convenience, the newly added content has been highlighted in the revised manuscript.

## Question 3

***Question:*** The use of the BMASS model for behavioral segmentation is a reasonable choice compared to traditional methods like change-point detection or entropy-based segmentation. However, no justification or sensitivity analysis is provided for this selection.

**Answer:** Thank you for this valuable suggestion. We agree that a thorough justification for the choice of the BMASS model, along with a sensitivity analysis, is essential for validating our methodology.

Our selection of BMASS is driven by the specific challenges of segmenting driving behavior, which manifests as high-dimensional, continuous time-series data. Traditional methods often struggle with such complexity. In contrast, the BMASS algorithm is particularly well-suited for this task for several key reasons:

**(1) *Multi-dimensional capability:*** It can natively handle multiple-dimensional time series simultaneously, which is crucial for capturing the holistic nature of driving behavior.

**(2) *Data-driven and robust***: As a data-driven approach, it does not rely on strong prior assumptions and has demonstrated robustness against the inherent fluctuations and noise present in real-world driving data.

**(3) *Proven applicability***: It has been successfully applied and validated in existing literature for driving behavior segmentation, establishing its credibility in this domain.

Regarding the sensitivity analysis, we recognize its importance in ensuring the robustness of our results. We have now performed a comprehensive sensitivity analysis on the key hyperparameters of the BMASS algorithm. The analysis confirms that our segmentation results are stable and not overly sensitive to minor variations in these parameters, thereby validating the reliability of our chosen configuration.

To incorporate these points, we have added a detailed justification of the BMASS selection and a summary of the sensitivity analysis to ***Section 3.1.*** The newly added content is highlighted in the manuscript.

## Question 4

***Question:*** The paper lacks a thorough discussion of its limitations and fails to provide concrete and actionable recommendations for future work.

**Answer:** Thank you very much for your constructive comments. We agree that the original manuscript required a more thorough discussion of its limitations and future work. In response to your feedback, we have made the following targeted revisions:

**(1) Dataset aspect**

We recognize that a primary limitation of our current study lies in the diversity of the dataset. To fully validate the generalizability and robustness of our proposed method, a wider range of driving scenarios is necessary. To address this, we have added a new **subsection 6.2.1**. Based on the design principles of our data collection experiments in Section 2.1, subsection 6.2.1 provides a detailed analysis of the current dataset’s limitations and outlines a concrete plan for future data augmentation.

**(2) Application aspect**

To address the lack of concrete recommendations for future work, we have added a new **subsection 6.2.2** dedicated to exploring the potential applications based on the extracted behavior primitives. This section elaborates on how these primitives can be utilized in specific areas, such as driver-centric behavior analysis and coaching, interactive behavior analysis with surrounding vehicles, and scenario generation for ADAS/AV testing. We have also included relevant references to provide a clear and actionable roadmap for subsequent research.

Thank you again for your valuable guidance. These revisions have significantly enhanced the completeness and forward-looking perspective of our paper.

## Question 5

***Question:*** Although the result analysis is numerically sound, it remains shallow in behavioral interpretation. The influence of each behavioral cluster on driver behavior and its application to decision-making systems should be further discussed.

**Answer**: Thank you very much for your insightful comment. We agree that the original manuscript lacked depth in its behavioral interpretation and did not sufficiently discuss applications on primitives. Guided by your feedback, we have made the following revisions:

**(1) On deepening the behavioral interpretation**

The initial semantic interpretation of the primitives relied solely on their statistical characteristics. We have now incorporated the context of the driving environment and the driver’s task to conduct a more in-depth analysis on each behavioral segments cluster, thereby assigning them more accurate semantic definitions. This content has been highlighted in **Section 5.2.2**.

**(2) On the application of behavior primitives**

We recognized that the initial draft’s lack of a detailed description of the primitives’ applications limited the reader’s ability to grasp their full potential. Therefore, we have added a dedicated subsection in **Section 6.2.2** that elaborates on the application of behavior primitives in areas such as decision-making systems. We have also included relevant references to clearly illustrate their application prospects for the reader.

Thank you again for your valuable feedback. These revisions have significantly enhanced the depth and practical utility of our paper.

## Question 6

***Question:*** The behavioral clusters extracted using VC-LDA are labeled but not semantically interpreted. Their real-world behavioral significance is not addressed.

**Answer:** We sincerely thank you for your valuable comment, which prompts us to re-examine our work from a deeper perspective. Your comment, along with **comments #11 and #13**, collectively inspired a fundamental revision of the semantic interpretation section for the driving behavior primitives.

We have moved beyond a purely statistical description and established a new, comprehensive analytical framework. This framework synthesizes surface-level statistical features with deeper contextual factors, including the driving environment and the driver’s intent or primary task. Following a structured process of ***Observation, Mechanism Analysis, and Semantic Definition,*** we have conducted an in-depth semantic interpretation of all five primitives.

Through this approach, we have endowed each primitive with a clear, real-world behavioral significance. The detailed analysis and results are now comprehensively revised and presented in ***Section 5.2.2*** of the revised manuscript, with all changes highlighted. This revision also fully addresses the concerns raised in Question #11 and #13. A more delicate analysis process is shown in the answer to Question #11.

## Question 7

***Question:*** The modeling of driver expectations is unclear — it is not specified whether these expectations are based on geographic context, driver experience, or environmental conditions.

**Answer**: Thank you for pointing out the need for greater clarity regarding the driver expectation model. We agree that this is a crucial point.

In our framework, driver expectation is a composite construct. It is determined at a cognitive level, where the driver makes a judgment about the immediate driving environment. This judgment is influenced by three key elements: the geographic/environmental context, the driver’s accumulated experience, and their personal driving style.

Once this expectation is formed, it informs the driver’s tactical decisions. The performance goals, such as safety, economy, efficiency, and comfort, are then evaluated and weighted according to the established expectation, leading to a final driving action based on a distinct set of criteria.

To better illustrate this process, we have expanded ***Section 3.1*** in the manuscript. It now includes a dedicated description of the expectation formation mechanism and the theoretical model that underpins our assumptions.

## Question 8

***Question:*** The potential applications of this research in AV and ADAS development, driver monitoring, driving education, and intelligent fleet planning are valuable. However, the paper lacks a direct and explicit discussion linking its findings to real-world industrial deployment.

**Answer**: Thank you for your insightful comment. We agree that the original manuscript lacked a direct and explicit discussion linking its findings to real-world industrial deployment. To address this valuable feedback, we have added **a new subsection, 6.2.2**, dedicated to this topic. In this section, we explicitly discuss the application of behavior primitives on driver-centric behavior analysis and coaching, interactive behavior analysis with surrounding vehicles, scenario generation for ADAS/AV testing. We have also included relevant references to support its industrial relevance. We believe this addition significantly strengthens the paper’s practical impact and value.

## Question 9

***Question:*** The sample size is limited to a small number of drivers, and the driving scenarios lack sufficient diversity.

**Answer**: Thank you for raising this important point regarding sample size and scenario diversity, which directly relates to the generalizability of our findings.

We acknowledge that our study is constrained by a limited sample size and a single driving scenario, primarily due to the practical costs associated with data collection. Consequently, we adopted a controlled experimental design: we held the driving scenario constant to focus on isolating the behavioral differences among drivers with varying levels of experience and driving styles. It is worth noting that the controlled scenario was not trivial; it was a complex 21.7 km route featuring 11 curves, sparse surrounding traffic, and specific triggered events.

This design choice was deliberate, prioritizing the internal validity of our study to effectively validate the proposed primitive extraction methodology. We recognize that this comes at the cost of some external validity, limiting the direct generalizability of our specific results.

To address this, we have added a detailed illustration of our experimental design philosophy to ***Section 2.1*** of the revised manuscript. This addition, which has been highlighted, clarifies the study’s limitations and emphasizes that the proposed methodology itself is generalizable to more diverse scenarios. Furthermore, expanding the diversity of driving scenarios is a key objective for our future research, which is illustrated in the added ***Section 6.2.1***.

## Question 10

***Question:*** The model does not account for exogenous variables such as road conditions, weather, or traffic density, which significantly affect driver behavior.

**Answer**: Thank you for this insightful comment, which further builds upon the discussion regarding the scope of our study.

We agree that exogenous variables such as road conditions, weather, and traffic density significantly influence driver behavior. To address the practical challenges of data collection and to focus on validating our core methodology, we adopted a controlled experimental design. In the simulator, we established a consistent scenario with specific road geometry, sparse traffic flow and specified traffic events for all sixteen participants. This approach was deliberately chosen to isolate the impact of driver-specific characteristics on the resulting behavior primitives.

We acknowledge that weather conditions were not included in this phase of the research, and we recognize this as a limitation of the current study. The impact of weather (e.g., rain, snow, fog) is a critical factor that we plan to incorporate in our future work.

It is important to emphasize that our proposed methodology is inherently extensible. Our subsequent research will focus on enriching the dataset with diverse weather conditions and more complex traffic scenarios to develop a model with greater generalizability and robustness. We have added a discussion of these future directions in ***Section 6.2.1*** of the manuscript and highlighted it.

## Question 11

***Question:*** The cluster interpretation remains general, lacking causal or behavioral depth. For example, what justifies the classification of a cluster as high-risk or defensive driving?

**Answer:** We sincerely thank the reviewer for these insightful and constructive comments. We fully agree that our initial interpretation of the clusters was too general and lacked the required causal, behavioral, and semantic depth. Your point about the need for functional and semantic analysis was particularly helpful and has guided us in a significant revision of this section.

To address these concerns, we have completely overhauled our analysis framework for interpreting the behavioral primitives. We have adopted a new, three-step interpretative structure for each cluster:

***Observation:*** A brief summary of the key statistical features.

***Behavioral & Contextual Interpretation:*** An in-depth analysis that connects the statistical features to the likely driving scenario (e.g., highway cruising, congested traffic) and the driver’s primary task or intent (e.g., maintaining speed, reacting to a hazard). This step directly addresses the need for causal and behavioral depth.

***Semantic Definition:*** A concise, functionally meaningful label that captures the essence of the behavior (e.g., “Reactive Emergency Braking”, “Cautious Lane-Changing”). This step provides the required functional and semantic analysis.

Crucially, we have enriched our analysis by incorporating the environmental context and the driver’s task, which were previously underutilized. For example, for what we now define as “Reactive Emergency Braking” (Cluster 4), we now explain that the high negative acceleration and jerk are not just data points, but indicators of a safety-critical, reactive maneuver triggered by an unexpected event, thus providing a clear functional and semantic justification for the label.

Following this new framework, the descriptions for all five clusters have been completely rewritten to provide the depth you requested. These revisions can be found in ***Section 5.2.2*** of the revised manuscript. All changes have been highlighted for your convenience.

We believe that these revisions now provide a much richer, more meaningful, and better-justified interpretation of the extracted driving behavior primitives, significantly strengthening the contribution of our work. Thank you again for your valuable guidance.

## Question 12

***Question:*** No sensitivity analysis is conducted on key model parameters (e.g., number of clusters, window size, discretization rate), limiting understanding of model robustness.

**Answer**: Thank you for your valuable feedback regarding the sensitivity analysis. We agree that sensitivity analysis is crucial for validating the model’s robustness.

Upon reflection, we realize that while the sensitivity analyses were conducted, they were integrated into different sections and were not explicitly labeled as “sensitivity analysis”. This understandably made them difficult to notice and created a hurdle for the reader’s comprehension.

Thanks to your valuable suggestion, we have carefully revised the manuscript. We have now explicitly point this issue to the relevant sections to significantly improve clarity and guide the reader.

The analyses are structured around the two main stages of our methodology: time-series behavior segmentation and segment clustering. For your convenience, we have listed the specific locations of these analyses below:

**(1) For the Time-Series Segmentation (Section 5.1 & 5.3)**

***Window Size Sensitivity:*** The impact of the time window size on segmentation quality is analyzed. Using segment length and the distribution of segmentation points as metrics, the optimal window size is identified. This analysis is presented in Section 5.1.

***Feature Space Sensitivity:*** The model’s sensitivity to the choice of input features by comparing a basic feature set with our proposed multi-type feature space is assessed. The results, which show superior performance and more accurate change-point detection with the multi-type space, are detailed in Section 5.3.

**(2) For the Segment Clustering (Section 5.2 & 5.4)**

***Number of Clusters Sensitivity:*** the optimal number of clusters by using perplexity as the evaluation metric is determined. This process is described in Section 5.2.

***Model Robustness:*** To ensure the robustness of the VC-LDA clustering approach, the VC-LDA model is compared with the conventional GMM-LDA. The comparison, based on inter-cluster distance, demonstrates that VC-LDA produces more distinct and well-separated clusters, confirming its effectiveness. Furthermore, we have supplemented the manuscript with two new figures that visualize the results of the driving behavior discretization. These figures are included to allow readers to directly observe the superiority of our proposed data discretization method, highlighting its effectiveness by accounting for both vehicle dynamics and the coupling characteristics between variables. This comparative analysis is provided in Section 5.4.

## Question 13

***Question:*** The extracted behavioral primitives are labeled but not functionally or semantically analyzed (e.g., what decision does a given cluster correspond to? Does it indicate responsiveness or aggressiveness?).

**Answer:** Thank you very much for pointing out this issue. The functional and semantic analysis of the primitives is a very important point in this paper. Our previous physical definitions of the primitives, based solely on their statistical features, were indeed too general.

Inspired by your profound analysis and the suggested direction for a solution, we have now conducted a comprehensive analysis for each primitive. This new approach follows the steps of: observing statistical features, considering the driving environment and the driver’s task that drive the primitive, and then providing a semantic interpretation. This allows us to define each primitive by integrating a deep-level, environment- and task-driven analysis with a surface-level statistical manifestation.

The relevant revisions have been made in ***Section 5.2.2*** and are highlighted.

## Question 14

***Question:*** The methodology of the VC-LDA model lacks formal mathematical description, including assumptions, inference procedure, and coupling formulation.

**Answer**: Thank you for your insightful comment. We agree that the methodology of the VC-LDA model required a more formal mathematical description, including its assumptions, inference procedure, and the formulation of its coupling mechanism.

To address this, we have restructured and clarified the description of the VC-LDA model in the revised manuscript. We now explicitly define VC-LDA as a two-stage framework and have elaborated on its mathematical foundations:

***Stage 1: Variable-coupling discretization.***

This is the core contribution of our work. It addresses the limitations of standard discretization methods by incorporating the complex, nonlinear, and time-lagged coupling relationships among features. The “coupling formulation” is not embedded within the LDA’s probabilistic model itself but is instead used as prior knowledge to guide the discretization process. The theoretical basis for this coupling is twofold:

(1) The nonlinear dynamics among basic features (velocity, accelerations, jerk) are governed by a simplified 3-DOF vehicle dynamics model.
(2) The temporal asynchrony between basic and constructed features arises from the driver’s decision-making hierarchy (i.e., the time lag between tactical expectations and operational control).

***Stage 2: Standard LDA Clustering.***

After obtaining the physically meaningful and smoothly transitioning discrete symbols from Stage 1, we apply a standard Latent Dirichlet Allocation model for clustering. The assumptions and inference procedure for this stage follow the well-established LDA framework (e.g., each driving segment is a mixture of behavior primitives, and each primitive is a distribution over discrete symbols). The key is that our novel discretization ensures the input to this standard model is of high quality and physically interpretable.

In summary, the novelty of VC-LDA lies in its sophisticated pre-processing (Stage 1), which makes the subsequent standard LDA clustering (Stage 2) effective and meaningful.

We have added a detailed breakdown of these two stages, the explicit model assumptions, and the theoretical foundation for the coupling mechanism in ***Section 4.1*** of the revised manuscript, with all changes highlighted for your convenience.

## Question 15

***Question:*** No mention is made of participant consent or ethical approval, which is essential for studies involving human subjects — particularly for journals that adhere to strict ethical review standards.

**Answer**: Thank you for raising this important point regarding ethical approval and participant consent. We agree that this is a crucial aspect of studies involving human subjects. In response, we have added a detailed statement to the ***Section 2.1*** of the manuscript, which reads:

*Participants were recruited from the general public. The recruitment criteria required that all individuals hold a valid driver’s license and have driven at least one hour per day over the past three months to ensure adequate driving familiarity and stable driving behavior. The study protocol was approved by the Ergonomics Research Ethics Committee of Transportation College, Jilin University. Prior to the experiment, all participants were fully informed of the research objectives, potential risks and benefits, and the intended usage of the collected driving data. Only driving behavior data is used in, no personally identifiable information is disclosed. Written informed consent was obtained from each participant.*

This addition has been highlighted in the manuscript for your convenience.

# Reviewer #2

## Question 1

***Question:*** The paper employs simulated driving data (RADS 8 DOF), which is reasonable. However, the sample size is small (only 16 drivers), and it is unclear whether the dataset covers diverse driving scenarios (e.g., urban, highway). The authors are encouraged to provide more details about data diversity or to discuss the generalizability of the approach.

**Answer:** Thank you for raising this important point regarding sample size and scenario diversity, which directly relates to the generalizability of our findings.

Driving behavior primitives are defined as the minimal, physically meaningful segments of driving behavior generated by a driver when facing diverse scenarios, including static road environments, weather conditions, and dynamic traffic conditions. These primitives are influenced by the scenario itself, as well as the driver’s experience and style.

To address the practical challenges of data collection and to focus on validating our core methodology, we adopted a controlled experimental design. This involved holding the driving scenario constant to isolate the behavioral differences among drivers with varying experience and styles. And the driving scenario is a complex 21.7 km route with 11 curves, sparse traffic, and specific triggered events. This deliberate choice prioritizes the internal validity of our study, allowing us to effectively validate the proposed primitive extraction method. We recognize that this comes at the cost of some external validity, which limits the direct generalizability of our specific results.

To clarify this design philosophy and its implications, we have added a detailed discussion to ***Section 2.1*** of the revised manuscript. This addition clarifies the study’s limitations and emphasizes that the proposed methodology itself is generalizable to more diverse scenarios.

Looking ahead, expanding the diversity of driving scenarios is a key objective for our future research. Our subsequent work will focus on enriching the dataset with diverse weather conditions and more complex traffic scenarios to develop a model with greater generalizability and robustness. We have added a discussion of these future directions in ***Section 6.2.1*** of the manuscript and highlighted it.

## Question 2

***Question:*** Some terms are used inconsistently, such as “multi-type variable space” and “multivariate space”, which appear interchangeably. It is recommended to unify terminology throughout the paper to avoid confusion.

**Answer:** Thank you for this insightful comment and for bringing the terminology inconsistency to our attention. In our manuscript, the term “multi-type variable space” was specifically chosen to emphasize that the BMASS segmentation method utilizes features from two distinct categories: the basic feature space and the constructed feature space. After careful consideration, we contend that “multi-type feature space” is a more appropriate term than “multi-type variable space.” The term “feature” more accurately represents the engineered criteria used for judgment, whereas “variable” can refer to raw, unprocessed data. This makes “feature” a more universal and precise descriptor within the context of our methodology. We agree that “multivariate space”, which is a more general statistical term, does not accurately convey this specific meaning. Following your recommendation, we have revised the manuscript to use “multi-type feature space” consistently throughout, and all instances of “multivariate space” have been replaced accordingly.

## Question 3

***Question:***  Lines 234-248 describe the process of time window selection, but the justification for ultimately adopting a 1-second window is not clearly or convincingly explained. The reasoning appears to involve logical gaps and inconsistent standards. This section would benefit from additional theoretical or experimental support.

**Answer**: Thank you for your insightful comment. We agree that the justification for selecting the 1-second window required greater clarity and a stronger theoretical foundation.

The selection of the optimal sliding window size is a critical step, as it directly impacts the construction of the multi-type feature space and, consequently, the quality of driving behavior segmentation. To address this, we have established a two-stage screening process to determine the optimal window size:

***Stage 1: Screening based on primitive duration.***

Our core premise is that driving behavior primitives, being the minimal segments with physical meaning, should not have excessive durations. Therefore, we used the average duration of lane change (6 seconds) as a benchmark. If a particular window size resulted in a high proportion of overly long primitives (e.g., approaching or exceeding 6 seconds), it was considered inconsistent with the “minimal” definition of a primitive. This provides our first quantitative criterion based on physical significance.

***Stage 2: Screening based on the distribution of segmentation points.***

Our previous justification for this stage was primarily qualitative and lacked theoretical rigor. Following your valuable suggestion, we have significantly revised this section. We now analyze the distribution of the relative positions of segmentation points and introduce two quantitative metrics, ***the width and flatness of the top region***, to screen the window sizes. This enhancement transforms our previous qualitative observation into a robust, quantifiable theoretical basis, significantly strengthening the argument.

By integrating the results from both stages, the 1-second window was ultimately adopted because it best satisfies the physical meaning of a “minimal segment” while its segmentation point distribution exhibits optimal stability and concentration.

The detailed analysis and corresponding modifications have been added to ***Section 5.1*** of the revised manuscript and highlighted for your convenience.

## Question 4

***Question:*** The references are somewhat outdated, with limited coverage of the last five years. It is suggested to add more recent studies, especially those focusing on driving behavior analysis, sequence segmentation, and clustering methods, to highlight the paper’s connection with the state of the art.

**Answer:** We sincerely thank the reviewer for this valuable suggestion. We agree that incorporating recent literature is crucial for positioning our work within the current state of the art.

In response, we have completely revised the Introduction Section. We have integrated numerous recent publications, particularly from the last five years, with a special focus on advancements in driving behavior analysis, sequence segmentation, and clustering methods. This revision not only updates the bibliography but also strengthens the manuscript’s motivation and clarifies its contribution relative to contemporary research.

For ease of review, the revised Introduction has been highlighted in the manuscript.
